# Umbilical cord blood hematological parameters reference interval for newborns from Addis Ababa, Ethiopia

**DOI:** 10.1186/s12887-021-02722-z

**Published:** 2021-06-11

**Authors:** Ammanuel Angelo, Girma Derbie, Asrat Demtse, Aster Tsegaye

**Affiliations:** 1 Department of Medical Laboratory, St Peter Specialized Hospital, Addis Ababa, Ethiopia; 2 Department of Medical Laboratory Sciences, College of Health Sciences, Addis Ababa University, Addis Ababa, Ethiopia; 3 Department of Obstetrics and Gynecology, St Peter Specialized Hospital, Addis Ababa, Ethiopia; 4 Department of Pediatrics and Child Health, School of Medicine/Tikur Anbessa Specialized Hospital, College of Health Sciences, Addis Ababa University, Addis Ababa, Ethiopia

**Keywords:** CBC, Reference interval, Umbilical Cord Blood, Neonates, Ethiopia

## Abstract

**Background:**

Several factors like altitude, age, sex, pregnancy, socioeconomic status, life style and race influence hematological reference interval (RIs), which are critical to support clinical decisions and to interpret laboratory data in research. Currently there are no well-established RIs for cord blood hematological parameters of newborns in Ethiopia. This study aims to generate RIs for umbilical cord blood hematological parameters of newborns from Addis Ababa, Ethiopia.

**Method:**

A cross-sectional study was conducted from January 1 to March 31, 2019 on healthy, term newborns (37–42 weeks) with normal birth weight born to apparently healthy pregnant mothers who had met the eligibility criteria. From 139 newborns, 2-3ml cord blood was immediately collected from the clumped cord using EDTA tube. The samples were analyzed using Sysmex KX 21 hematology analyzer. Data was entered and the 2.5th and 97.5th percentiles (upper and lower reference limit) were determined using non parametric method by SPSS version 23. The non-parametric independent Mann-Whitney U test (Wilcoxon rank-sum test) was used to compare the distribution of the parameters between genders, modes of deliveries and gestational age. *P* value less than 0.05 was considered to declare statistical significance.

**Result:**

The median values and 95 % reference interval for umbilical cord blood hematological parameters of newborns were as follows: WBC = 12.4 [6.6–19.4] x10^9^/L, RBC = 4.51 [3.55–5.52] x10^12^/L, HGB = 15.8 [12.4–19.7] g/dL, HCT = 45.9[37.9–56.3]%, MCV = 102.1[83.9-111.6] fL, MCH = 35.3 [29.4–39.1] pg, MCHC = 34.3 [32.3–37.4] %, PLT = 236 [146–438] x10^9^/L, LYM = 37.5 [16.6–63.0] %, MXD = 7.9[1.7–15.8] %, NEU = 53.7[30.3–78.4] %, RDW = 15.6[12.0–19.0]%, PDW = 11.0[9.1–15.7]% and MPV = 9.4[8.1–11.8] fL. The current study found no significant difference between genders, except RDW (*P* = 0.01), and gestational age group, but there was significant difference for WBC (*p* = 0.007), RBC (*p* = 0.018) and Absolute NEU (*p* = 0.001) by delivery type where newborns delivered through caesarean section had lower values for these three parameters compared to those with spontaneous delivery.

**Conclusions:**

hematological reference intervals in cord blood were established for the first time from healthy newborns of Addis Ababa and its surrounding. The values are applicable for newborns from this area. Larger study throughout the country is warranted.

## Introduction

Reference intervals (RIs) aid in the interpretation of laboratory data in patient management, clinical trials, and selection of eligible participants for vaccine trials [1]. These values are affected by several factors including age, sex, race, geographical location and dietary pattern [2]. A wealth of published studies demonstrated the age related changes in hematological parameters. Neonates exhibit profound quantitative as well as qualitative hematologic differences compared to older children and adults [3]. At birth, hematological parameters of term newborns are significantly higher than those of older children and adults [4, 5]. Thus, it is inappropriate to use adult reference ranges for the assessment of pediatric blood [6, 7].

On the other hand, utilizing a reference interval obtained from somewhere else to a population of interest could potentially lead to inappropriate patient management and unnecessary use of resources [8, 9]. Cord blood hematological parameters reference intervals are vital in neonatal care and transplantation medicine [10, 11]. Cord blood is a blood collected from long and helical cord that connects the fetus with the mother for substances exchange [12]. Establishing normal neonatal reference interval has been difficult because of drawing blood from healthy neonates. Challenges of getting ethical approval for this vulnerable group and consent from the mothers also explain why limited RIs established from cord blood. Moreover, apart from the neonates other maternal factors are also needed to be considered [13–16].

According to different studies, maternal anemia had effect on cord blood hemoglobin and newborn weight [17–20], although it has been reported that routine hematological values of newborns are independent to that of maternal hematological values [21, 22]. A likely explanation given by those stating the dependency on maternal status is the high micronutrient intake inadequacy during pregnancy [23–25]. Maternal factors like smoking habit [26], heavily drinking alcohol [27], medical problems like diabetic mellitus, eclampsia, hypertension [28–30] and mode of delivery and frequency of pregnancy affect the hematological profile of neonates [31–33]. Delayed cord clamping and umbilical cord milking also affect hematological parameters [34, 35]. Otherwise, cord blood collection is a safe way to collect sample compared to cannula and other vascular catheters which cause thrombophlebitis, infection, and extravasation [36]. Thus, in the current study we used cord blood collection method.

In spite of the fact that reference intervals can play an important role in guiding the assessment of hematological changes in neonatal care, there is no published reference interval for hematological parameters in cord blood of Ethiopians except some earlier efforts from Central Ethiopia [37] and recently from Northwest [38]. This study aimed at providing such intervals for cord blood samples from Addis Ababa.

## Materials and methods

### Study design and setting

A cross-sectional study was conducted from January 1 to March 31, 2019 in Addis Ababa, Ethiopia. Addis Ababa is the capital city of Ethiopia and seat for African Union, located at an elevation of about 2440 m (about 8000 ft) above sea level. Study participants were recruited from mothers coming to St Peter Specialized Hospital to get delivery service. The hospital is one of the five Federal Hospitals under the Ministry of Health which is located in the beautiful mountainous *Entoto* area. It used to be a Tuberculosis (TB) Specialized Hospital for many decades and expanded its service in recent years to provide comprehensive service to the needy residents including maternal and child care. The hospital performs about 3600 deliveries annually.

### Sample size determination and sampling Technique

A total of 139 newborns delivered in St. Peter Specialized Hospital were included in this study using convenient non-probability sampling technique. As per the Clinical Laboratory Standard Institute (CLSI) guideline, about 120 samples per partition is required to determine the 95 % reference interval [2]. Employing the *a priori* selection method, eligible volunteering mothers aged 18 to 45 years were recruited in the study. Mothers with the following conditions were excluded: those with medical conditions like infectious (e.g. Hepatitis B, HIV, Syphilis), chronic illness (e.g. Insulin-dependent diabetes mellitus), obstetric (e.g. less than six months from abortion, preeclampsia), psychological problems and social habits (e.g. smoking, heavily alcohol drinking). Comprehensive techniques including diagnostic tests (laboratory tests, ultrasound) and history from their hospital card extracted using structured format were used to exclude mothers with the listed conditions. On the other hand, mothers who had Hgb > = 11.0 g/dL [39] and inter-pregnancy interval of more than or exactly 18 months as per WHO recommendation [40] were included in the study. Whereas, *posteriori* selection method was used to include eligible newborns who were term (37–42 weeks), had 5th minute Apgar score of > = 7 and birth weight within 2.5-4 Kg. Babies with respiratory distress, meconium staining, gross congenital anomalies, umbilical cord with true knot, and babies delivered by instrumental delivery were excluded.

### Data Collection Procedure

Prior to data collection, all the professionals who participated in data collection were oriented about the aim of the study, selection of participants, data confidentiality, safety precautions during collection, transportation, and storage of cord blood samples. Predesigned questionnaire was used to collect demographic information and a brief medical history from consenting eligible mothers. As delivery is stressful event, all mothers were asked for an informed signed consent and answer the questionnaire by the help of the attending midwives before entering the labor room. The umbilical cord was clamped in at least 1 min after birth and 2–3 ml of cord blood was collected by midwives or operation room (OR) nurse using EDTA tube. The sample was well mixed (8-10x) and immediately transported to hematology laboratory for analysis.

### Screening Tests

Hepatitis B surface antigen, HIV and syphilis antibody tests are routinely performed for pregnant women as part of their antennal care (ANC) follow up. Ultrasonography test was also used to rule out fetal gross congenital anomalies. These data were used to select eligible mothers.

### Hematological Analysis

Complete blood count (CBC) namely white blood cell (WBC), Diff count (neutrophil count (NEU#), lymphocyte count (LYM#), mixed cells count (MXD#), neutrophil percentages (NEU%), lymphocyte percentages (LYM%), mixed cells percentages (MXD%)) red blood cells (RBC), hemoglobin (HGB), hematocrit (HCT), mean corpuscular volume (MCV), mean corpuscular hemoglobin (MCH), mean corpuscular hemoglobin concentration (MCHC), red cell distribution width (RDW), platelets (PLT), mean platelet volume (MPV) and platelet distribution width (PDW) were analyzed using Sysmex KX-21 N (Sysmex Corporation, Kobe, Japan) [41] automated hematology analyzer. The analyzer performs 18 parameters within one minute. It utilizes three detector blocks for WBC (DC detection method), HGB (Non-cyanide HGB method), RBC and PLT (DC Detection method)). Peripheral blood smears from cord blood samples were prepared and stained using Wright’s stain for investigation of red blood cell morphology, white blood cell and platelets abnormalities.

### Quality Assurance

Cord blood was collected by experienced midwives or OR nurses following the guideline for cord blood sample collection. In addition, orientation was given on proper collection and handling of CB. Three level whole blood controls were used to ensure the quality of CBC using Sysmex KX-21 N. All procedures were performed following standard operating procedures (SOPs).

### Statistical Analysis

Data from the questionnaires and laboratory results were coded and checked for completeness. Data were then entered and analyzed using IBM SPSS-version 23 statistical software for windows. Following the CLSI guide, 2.5th and 97.5th percentiles for hematological parameters were calculated for 139 newborns of both genders. The non-parametric Mann-Whitney U test was used to compare the distribution of the parameters between genders, delivery modes (other than Instrument assisted delivery), and gestational age groups. Additionally descriptive statistics (minimum, maximum, mean, SD, median) were also determined. *P* value less than 0.05 was sued to declare statistical significance.

## Results

A total of 139 healthy full-term newborns consisting of 67 (47.9 %) males and 72(51.8 %) females were enrolled in the study. About 82.7 % of the mothers were in the age group 18–30 years, 15.8 % were from outside Addis Ababa (surrounding *Weredas*), 84.9 % were literate, 55.4 % unemployed, and 47.5 % (66/139) were having first time delivery (Table 1).

**Table 1:** Sociodemographic and clinical characteristics of study participants in Addis Ababa, Ethiopia, January 1 to March 30, 2020 (*n* = 139)

Information			Frequency	Percent
Age of Mother (Years)	18–30		115	82.7
	31–45		24	17.3
Residence	Addis Ababa		117	84.2
	Outside Addis Ababa^a^		22	15.8
Educational level	Illiterate		21	15.1
	Literate		118	84.9
Employment Status	Employed		62	44.6
	Unemployed		77	55.4
Marital Status	Married		133	95.7
	Other		6	4.3
Pregnancy History	Yes		73	52.5
	No		66	47.5
Pregnancy Interval (*n* = 73)^a^	1-1year & 6months		6	8.2
	>=2years		67	91.8
Delivery mode	Previously	SVD	56	76.7
		CS	17	23.3
	Currently	SVD	93	66.9
		CS	46	33.1
Sex of Baby	Male		67	48.2
	Female		72	51.8
Weight of Baby (kg)	2.5-3.0		74	53.2
	> 3		65	46.8
Gestational age (weeks)	37.0–39.0		60	43.2
	39.1–42.0		79	56.8

^a^these are special Weredas around Addis Ababa (Administrative divisions)

For all hematological parameters, there were no statistically significant difference by gestational age [37-39.1 versus 39.2–42 months] (*p* > 0.05). Independent Mann-Whitney U (Wilcoxon rank-sum) test between delivery modes shows significant difference (*p* < 0.05) of which newborns delivered through caesarean section (C/S) had lowered value for WBC (median with 95 % RIs) = 11.1[6.6–19.4], RBC = 4.39[3.55–5.52], and absolute NEU = 6.0[2.7–12.8] compared to newborns delivered through spontaneous vaginal delivery (SVD) with values for WBC = 12.9[6.6–19.4], RBC = 4.55[3.55–5.52], and absolute NEU = 7.6[2.7–12.8] (*p* < 0.05) (Table 2).

**Table 2:** Independent (2 Groups) Mann-Whitney U Test* of selected hematological parameters by gender, mode of delivery and gestational age

Parameters	*P*- value by sex	*P*-value by mode of delivery	*P*-value by gestational age
WBC(x10^9^/L)	0.787	**0.007**	0.366
RBC(x10^12^/L)	**0.075**	**0.018**	0.852
HGB (g/dL)	0.374	0.110	0.904
HCT (%)	0.861	0.193	0.707
MCV (fL)	0.171	0.117	0.990
MCH (Pg)	0.139	0.094	0.832
MCHC (g/L)	0.386	0.519	0.540
RDW-CV(%)	**0.01**	0.318	0.197
PLT (x10^9^/L)	0.369	0.291	0.855
LYM%	0.274	0.348	0.325
MXD%	0.807	0.666	0.915
NEU%	0.227	0.262	0.315
LYM#	0.188	0.420	0.781
MXD#	0.657	0.157	0.765
NEU#	0.684	**0.001**	0.264
PDW-CV(%)	0.199	0.296	0.188
MPV (fL)	0.913	0.858	0.503

*P values less than 0.05 determines significance Level

Sex specific and combined 2.5th and 97.5th percentile for complete blood count parameters from umbilical cord blood is summarized in Table 3. Statistically significant differences by sex were not detected for any of the parameters except RDW-CV in which females showed lower median value (*p* < 0.05) than males. The combined median and 95 % reference value of cord blood parameters as shown in Table 3 were for WBC = 12.4 × 10^9^/L [6.6–19.4], RBC = 4.51 × 10^12^/L [3.55–5.52], HGB = 15.8 g/dL [12.4–19.7], HCT = 45.9 % [37.9–56.3], MCV = 102.1fL [83.9-111.6], MCH = 35.3pg [29.4–39.1], MCHC = 34.3 % [32.3–37.4], PLT = 236 × 10^9^/L[146–438], %LYM = 37.5 % [16.6–63.0], %MXD = 7.9 % [1.7–15.8 ], and %NEU = 53.7 % [30.3–78].

**Table 3:** Reference Intervals for umbilical cord hematological parameters by Sex of Newborns from January 1 to March 31, 2019 G.C, Addis Ababa, Ethiopia (n = 139)

Parameter	Sex	N	Median	Min	Max	2.5	97.5	*P*-value
WBC (x10^9^/L)	M	67	12.6	6.5	19.6	6.6	19.3	0.787
	F	72	12.4	5.8	23.5	6.1	23.2	
	Combined	139	12.4	5.80	23.5	6.6	19.4	
RBC (x10^12^/L)	M	67	4.59	3.54	5.70	3.59	5.59	0.075
	F	72	4.44	3.10	5.70	3.37	5.30	
	Combined	139	4.51	3.10	5.70	3.55	5.52	
HGB (g/dL)	M	67	15.9	12.3	20.0	12.4	19.8	0.374
	F	72	15.7	10.8	20.6	11.1	18.8	
	Combined	139	15.8	10.8	20.6	12.4	19.7	
HCT (%)	M	67	46.0	36.0	60.2	37.9	57.8	0.861
	F	72	45.45	36.4	61.8	37.3	56.3	
	Combined	139	45.9	36.0	61.8	37.9	56.3	
MCV (fL)	M	67	102.0	81.9	115.5	82.1	112.8	0.171
	F	72	102.3	85.4	123.3	88.5	113.5	
	Combined	139	102.1	81.9	123.3	83.9	111.6	
MCH (Pg)	M	67	35.1	28.6	39.7	28.8	39.4	0.139
	F	72	35.3	27.8	41.1	31.6	38.7	
	Combined	139	35.3	27.8	41.1	29.4	39.1	
MCHC (g/L)	M	67	34.6	32.6	37.5	32.8	37.2	0.386
	F	72	34.3	30.0	37.9	31.0	37.7	
	Combined	139	34.3	30.2	37.9	32.3	37.4	
PLT (x10^9^/L)	M	67	230.0	145.0	469.0	146.7	466.2	0.369
	F	72	241.5	141.0	433.0	146.0	418.2	
	Combined	139	236.0	141.0	469.0	146.0	438.0	
%LYM	M	67	36.6	4.8	71.2	10.8	64.6	0.274
	F	72	37.8	14.7	76.0	19.4	66.4	
	Combined	139	37.5	4.8	76.0	16.6	63.0	
%MXD	M	67	8.3	1.5	17.8	1.7	15.8	0.807
	F	72	7.85	0.0	17.3	0.00	16.6	
	Combined	139	7.9	0.0	17.8	1.7	15.8	
%NEU	M	67	54.7	22.5	88.4	26.9	84.1	0.227
	F	72	53.4	18.6	82.3	29.7	75.4	
	Combined	139	53.7	18.6	88.4	30.3	78.4	
#LYM	M	67	4.4	0.8	7.7	1.9	7.7	0.188
	F	72	4.6	1.7	10.6	1.9	10.4	
	Combined	139	4.5	0.8	10.6	1.9	8.3	
#MXD	M	67	1.0	0.0	2.4	0.1	2.3	0.657
	F	72	1.0	0.0	2.5	0.0	2.4	
	Combined	139	1.0	0.0	2.5	0.1	2.4	
#NEU	M	67	6.8	1.9	15.7	2.0	15.4	0.684
	F	72	6.3	2.5	11.2	2.8	10.8	
	Combined	139	6.6	1.9	15.7	2.7	12.9	
RDW-CV (%)	M	67	16.0	12.9	18.3	13.0	18.3	0.01
	F	72	15.0	12.0	19.0	12.0	19.0	
	Combined	139	15.6	12.0	19.0	12.0	19.0	
PDW-CV (%)	M	67	11.2	9.1	16.5	9.3	16.2	0.199
	F	72	10.8	8.6	15.8	8.6	14.8	
	Combined	139	11.0	8.6	16.5	9.1	15.7	
MPV(fL)	M	67	9.4	7.0	11.3	7.4	11.2	0.913
	F	72	9.35	8.0	12.5	8.1	12.3	
	Combined	139	9.4	7.0	12.5	8.1	11.8

**Table 4:** Mean ± SD or Median Comparison with company derived values and other published reference values

Parameters	RI current study			Sysmex KX 21 [41]	Sudan [42]	Nigeria [43]	Saudi Arabia [44]	Pakistan [45]
	Median	Mean ± SD	95 % RI	RI	Mean ± SD	Mean ± SD	Median	Mean ± SD
WBC	12.4	12.4 ± 3.38	6.6–19.4	9.0–30.0	12.3 ± 4.17	13.1 ± 5.20	16.1	13.7 ± 4.00
RBC	4.51	4.51 ± 4.49	3.55–5.52	4.1–6.7	4.34 ± 0.60	4.05 ± 0.55	5.10	-
HGB	15.8	15.8 ± 1.64	12.4–19.7	15.0–24.0	14.4 ± 1.55	13.9 ± 1.50	17.7	15.4 ± 1.90
HCT	45.9	46.1 ± 4.62	37.9–56.3	44–70	44.1 ± 5.14	44.8 ± 5.78	53.2	-
MCV	102.1	101.2 ± 5.97	83.9-111.6	102–115	105.5 ± 5.14	110.4 ± 11.88	106	103.4 ± 4.60
MCH	35.3	35.1 ± 1.97	29.4–39.1	33.0–39.0	33.5 ± 1.99	32.6 ± 4.13	35.5	33.8 ± 1.60
MCHC	34.3	34.5 ± 1.17	32.3–37.4	32.0–36.0	33.1 ± 1.19	29.8 ± 1.64	33.2	-
RDW-CV	15.6	15.4 ± 1.60	12.0–19.0	11.8–15.6	19.8 ± 4.26	-	-	18.5 ± 18
PLT	236	245.5 ± 69.78	146.0-438.0	140–385	261 ± 83.16	225.1 ± 72.21	234	285 ± 62
LYM%	37.5	38.2 ± 10.96	16.6–63.0	-	-	-	-	-
MXD%	7.90	8.0 ± 3.44	1.7–15.8	-	-	-	-	-
NEU%	53.7	53.9 ± 10.84	30.3–78.4	-	-	-	-	-
#NEU	6.6	6.7 ± 2.42	2.7–12.9	6.0–26.0	-	-	-	7.7 ± 3.00
#LYM	4.5	4.7 ± 1.93	1.9–8.3	2.3–10.8	-	-	-	5.1 ± 1.80
#MXD	1.0	1.1 ± 0.88	0.1–2.4	0.1–3.6	-	-	-	-
PDW	11.0	11.6 ± 2.22	9.1–15.7	-	-	-	-	-
MPV	9.4	9.5 ± 0.90	8.1–11.8	-	-	-	-	-

*Units: WBC (x10^9^/L), RBC (x10^12^/L), HGB (g/dL), PLT (x10^9^/L), MCV (fL), MCH (pg), MCHC (g/L), RDW-CV (%)

The study also tried to compare the established cord blood RI with that provided by Sysmex for 0–24 h old newborns and other previous studies. Most studies present their findings as Mean ± SD and hence the comparison was made accordingly as described in Table 4.

## Discussion

This study aimed at providing reference intervals for selected hematological parameters from cord blood samples and compares the findings with company derived as well as other published reports [41–45]. Comparison of results according to sex, delivery modes and gestational age group were done. There was no statistically significant gender difference (p > 0.05) for all hematological parameters except RDW, which was a consistent finding with that of Greece [46]. The study from Greece, unlike the current study, has reported gender differences for WBC, NEU and PLT as well. Whereas, previous studies including from Korea [47], South India [48], Nepal [49], Saudi Arabia [44], Iran [50], Nigeria [43] and Sudan [42], on the other hand, concluded that there are no statistically significant differences by gender at this early life. Though the literature cannot provide consistent observations regarding gender difference in cord blood hematological parameters, the mechanisms remain to be well elucidated.

The current study demonstrated lower and narrowed RIs mainly for WBC, RBC, HGB, HCT and absolute 3 part differential counts (#NEU, #LYM, #MXD) parameters compared to reference value provided by Sysmex KX-21 hematology analyzer for newborns (0-24 h). Notably, the difference in the Neutrophils RI was remarkable (2.7–12.9 versus 6.0–26.0 × 10^9^/L, current versus Sysmex RIs, respectively) [41]. The consistency with a study from north Ethiopia, which reported a lower neutrophil count RI of 2.96–13.54 × 10^9^/L compared to the RI given by the company using the same machine Sysmex KX-21 N corroborated the notion that neutropenia is a common condition in Africans [51].

In the current study, RBC, HGB and HCT values were higher compared to Taiwan [33], Greece [46], Korea [47], South India [48], Pakistan [45], Nepal [49], Nigeria [43] and Sudan [42]. These might be due to the high altitude in Addis Ababa and mode of deliveries in which the majorly of participants in our study had spontaneous vaginal delivery [31–33]. Except the lower reference limit of HGB which is lower than the study from northern part of Ethiopia, the median and upper limit are consistent with a study from north Ethiopia [38], which is also a highland area (i.e., 12.4–19.7 (15.8 g/dL) current study vs. 13.32–19.64 (16.0 g/dL) from north Ethiopia).

MCV value in this study was surprisingly lower than most studies [33, 42–44, 46–50] including the study from north Ethiopia [38] and needs further investigation. RDW value in our finding, which is a marker of anisocytosis, was lower than Saudi Arabia [44], Iran [50] and Nigeria [43] but higher than that of Greece [46]. Nutritional variations could contribute to the observed differences [39].

In the current study, we found higher total WBC compared to studies from Taiwan [33], Greece [46], Korea [47], and Nepal [49]. This might be due to spontaneous vaginal delivery was the major mode of delivery in our country which affect the fetal hemogram [31–33]. However, the reference values from South India [48], Saudi Arabia [44], Pakistan [45], and Nigeria [43] are slightly higher than our finding. The current WBC finding was also lower compared to the limited published data from Ethiopia. The respective median and 95 % reference intervals were 12.4 (6.6–19.4) x10^9^/L in the current study versus 13.1 (7.0–20.9) x10^9^/L [37] from central Ethiopia using Coulter T 540 and 12.8 (7.64–22.16) x10^9^/L from north Ethiopia [38]. Of note, the value is much lower than the currently in use company derived RI which is 9.0–30.0 × 10^9^/L. Such inconsistencies underscore the need for locally established RIs for the target population.

The PLT value in the present study was higher than Taiwan [33], Korea [47], Nepal [49] and Nigeria [43]; however, it was lower than studies from South India [48], Pakistan [45], Saudi Arabia [44], Iran [50] and Sudan [42]. Compared to the study from Ethiopia, the PLT count was slightly higher (146.0-438.0 (236) x10^9^/L vs. 132.74–413.4 (276) x10^9^/L). Though technical differences in specimen handling and analysis [52] and cord clumping time [35] among the different studies cannot be ruled out, the current study has strictly followed protocols to ensure quality.

Physiological, nutritional and ethnic differences could also contribute to variations among populations, although the earlier study from Ethiopia favors environmental factors as a main cause [37]. Birth is also stressful event accompanied with hormone regulated inflammatory action which increases cell mobilization [53]; hence, differences in this event could cause differential movement of cells and perhaps partly contribute to differences seen among studies. The hematological reference intervals for Ethiopian cord blood, might play important role in neonatal care and transplantation medicine (stem cell transplantation from cord) to establish Umbilical Cord Blood Bank (UCBB). Cord blood hematological analysis as well as stem cell transplantation service is not part of the routine clinical care in the country. It is, thus, recommended that laboratories to incorporate cord blood analysis to their routine test lists and laboratory handbooks. In line of improving the neonatal clinical service and establishment of stem cell transplantation, implementation of such practices is not an option in this era of advancement in the clinical field.

## Conclusions

Since this study is pioneer of its kind with regards to selected hematological reference intervals in cord blood from Addis Ababa, the values obtained from our study could provide reference intervals for some hematological parameters in healthy newborns (0-24rs) of Addis Ababa and its surrounding special *weredas*. However, the results need to be confirmed by larger samples from different parts of the country for wider use.

## Data Availability

All data generated or analyzed during this study are included in this published article. The dataset can be available from the corresponding author on reasonable request.

## Abbreviations

RI: Reference interval
EDTA: Ethylenediaminetetraacetic acid
CBC: Complete Blood Count
WBC: White Blood Cell
RBC: Red Blood Cell
HGB: Hemoglobin
HCT: Hematocrit
MCV: Mean Cell Volume
MCH: Mean cell Hemoglobin
MCHC: Mean cell Hemoglobin Concentration
RDW-CV: Red Cell Distribution Width-Coefficient of variation
PLT: Platelet
LYM: Lymphocyte
MXD: mixed population of Monocytes Eosinophils and Basophils
NEU: Neutrophils
PDW: Platelet Distribution Width
MPV: Mean Platelet Volume
CS: Cesarean section
SVD: Spontaneous Vaginal Delivery

## Declarations

### Authors' information

A.A has a Masters in Clinical Laboratory Science with specialty in Hematology & Immunohematology from Department of Medical Laboratory Sciences (DMLS) of Addis Ababa University. A.T, PhD, Associate Professor in Hematology & Immunohematology Unit of DMLS, AAU; G.D is an MD OB-GY specialist at St Peter Hospital, A.D is an MD, Neonatologist in School of Medicine, AAU.
